# Perinatal staff perceptions of safety and quality in their service

**DOI:** 10.1186/s12913-014-0591-4

**Published:** 2014-11-28

**Authors:** Suzanne V Sinni, Euan M Wallace, Wendy M Cross

**Affiliations:** The Ritchie Centre, Department of Obstetrics and Gynaecology, Monash University and MIMR-PHI Institute of Medical Research, 246 Clayton Road, Clayton, Victoria 3168 Australia; School of Nursing and Midwifery, Monash University, Clayton, Victoria Australia

**Keywords:** Maternity care, Clinical governance, Midwifery dominance, Safety, Qualitative research

## Abstract

**Background:**

Ensuring safe and appropriate service delivery is central to a high quality maternity service. With this in mind, over recent years much attention has been given to the development of evidence-based clinical guidelines, staff education and risk reporting systems. Less attention has been given to assessing staff perceptions of a service’s safety and quality and what factors may influence that. In this study we set out to assess staff perceptions of safety and quality of a maternity service and to explore potential influences on service safety.

**Methods:**

The study was undertaken within a new low risk metropolitan maternity service in Victoria, Australia with a staffing profile comprising midwives (including students), neonatal nurses, specialist obstetricians, junior medical staff and clerical staff. In depth open-ended interviews using a semi-structured questionnaire were conducted with 23 staff involved in the delivery of perinatal care, including doctors, midwives, nurses, nursing and midwifery students, and clerical staff. Data were analyzed using naturalistic interpretive inquiry to identify emergent themes.

**Results:**

Staff unanimously reported that there were robust systems and processes in place to maintain safety and quality. Three major themes were apparent: (1) clinical governance, (2) dominance of midwives, (3) inter-professional relationships. Overall, there was a strong sense that, at least in this midwifery-led service, midwives had the greatest opportunity to be an influence, both positively and negatively, on the safe delivery of perinatal care. The importance of understanding team dynamics, particularly mutual respect, trust and staff cohesion, were identified as key issues for potential future service improvement.

**Conclusions:**

Senior staff, particularly midwives and neonatal nurses, play central roles in shaping team behaviors and attitudes that may affect the safety and quality of service delivery. We suggest that strategies targeting senior staff to enhance their performance in their roles, particularly in the training and teamwork role-modeling of the transitory junior workforce, are important for the development and maintenance of a high quality and safe maternity service.

## Background

Delivering safe, effective and appropriate care is clearly the aim of all maternity services. Over recent decades, with the aim of improving the quality and safety of delivered care, much attention has been paid to the development of evidence-based guidelines, staff education, risk management and reporting systems. However, perhaps less attention has been afforded to assessing and influencing staff attitudes and behaviours. This is important because patient safety is strongly influenced by the attitudes and behaviours of the clinicians delivering the care [1]. Teams displaying high levels of mutual respect, trust and cohesion are significantly more likely to consistently provide high quality care with lower rates of adverse events [2,3]. Outside of healthcare, private industry across diverse sectors, including manufacturing, finance, retail and hospitality, have long applied an awareness of the influence of team dynamics on productivity and safety to build capability and enhance outcomes [4-10].

In healthcare while the past few decades have seen increasing attention to safety and quality innovation much effort remain focussed on measuring health outcomes themselves [11-15]. While health outcome data are certainly important, and readily measurable, outcomes on their own do not provide information about the factors that may hinder optimal care delivery and thereby compromise outcomes [11,12]. In short, they do not provide insights into the processes that might underlie poor outcomes but rather simply identify that poor outcomes have occurred. Such an outcome-focused approach cannot directly inform what changes are required to effect improved care and better outcomes. Indeed, despite all the efforts around patient safety over decades of study overall rates of medical error have essentially remained constant [16-18].

However, more recently, clinical team behaviour has been investigated as a possible contributor to compromised patient safety and a focus on improving team dynamics developed as part of the pathway to improved outcomes [1-3]. It has been estimated that ineffective team communication is an important contributory factor in about 8 out of 10 adverse events [19]. Of all healthcare, there is a risk that the provision of maternity care, in particular, is structured in a manner that may undermine effective team behaviour and thereby impair outcomes. Specifically, maternity care involves two separate professional groups – midwives and obstetricians – that, at least in some countries such as Australia, New Zealand and the UK, are both independent and able to exert differing ideological practices, often for the same individual patient [20-28]. For example, in general, midwives are trained as skilled birth attendants to facilitate a normal physiological process whereas obstetricians are medically trained to expedite a condition fraught with risk requiring high levels of intervention [20-28]. We wondered whether the issues and potential tensions that these might bring to the dynamics of the attendant clinical team might impact upon safety. Accordingly, we set out to examine staff perceptions of safety and quality using established qualitative methodology, exploring issues of influence on team behaviours. Specifically, we aimed to assess how the workforce perceived the level of safety of their service, to explore perceived interactions between professional groups, and to identify issues that might undermine team performance and thereby put safety at risk.

## Methods

### Setting

The study was conducted within a new maternity service established for women with a low risk pregnancy in a recently built hospital. The service was a planned expansion of an existing maternity program that already provided secondary and tertiary maternity services at the two other established hospitals within the organization (Monash Health). At the time of the study there were 1000 births per annum in the low risk service that was the focus of the study. The service offered two models of care: midwifery-led or shared antenatal care between midwives and community based General Practitioners (GPs). In both models of care, intrapartum care was primarily provided by midwives with the support of on-site junior resident medical officers and on-call specialist (consultant) obstetricians. All staff were expected to follow existing organizational protocols relating the management of pregnancy and labour with clear and robust criteria for consultation and referral to medical care when deviations from normal were present.

### Participants

All hospital-based staff involved in perinatal care and/or its administration were invited to participate. Invitations to participate in the study were extended at a regularly scheduled work meeting then distributed via electronic mail. Twenty-three staff were interviewed, comprising a self-selected but representative cross section of the workforce. Table 1 summarizes the participants and their levels of experience. Data saturation, as evidenced by no new emerging themes, was achieved after 15 interviews. However, all who agreed to participate were offered an interview and data were extracted from all 23 interview transcripts. No participant withdrew.

**Table 1:** **The role and numbers of participants**

**Staff role**	**Number interviewed**
Midwives	10 (8 senior midwives*, 1 junior midwife, 1 postgraduate midwifery student)
Obstetric staff	6 (5 consultant obstetricians, 1 junior medical officer)
Nurses	4 (2 senior neonatal nurses*, 2 nurse coordinators)
Paediatricians	2 (1 consultant paediatrician, 1 in a paediatric training program)
Clerical staff	1

*Senior midwives and neonatal nurses include managers, associate managers and clinical midwife/nurse specialists.

### Data collection

We used naturalistic interpretive design to inform a deep understanding of the subject matter [29]. This approach was applicable because two of the researchers (SVS, EMW) were involved in the planning and implementation of the maternity service and were responsible for overseeing quality and safety of the service and so were known to stakeholders. In the first phase of the study, staff focus groups [30] were established to engage the staff and to appraise them of the purpose of the study. Questions from a validated patient safety culture survey [31] were modified to form the basis of a semi-structured questionnaire that was then used to generate open-ended discussions during in-depth interviews about the safety and quality of the service (Table 2). Semi-structured interviews included funnelling by beginning with open ended questions and drilling down to points of interest, story telling to encourage the interviewee to elaborate by recounting their personal experiences, probing where the interviewer sought clarification, and paraphrasing by repeating back what the interviewee said to ensure common understanding to prompt further discussion [30]. The interview protocol was designed to maximize consistency including details about the interview such as time, place and person being interviewed, instructions for the interviewer and questions that have a logical sequence with ample opportunity for the interviewee to elaborate [32].

**Table 2:** **Interview Questions**

1	*What are your experiences of the safety and quality of the maternity service?*
2	*Does evidence inform clinical practice decisions at the maternity service?*
3	*What happens when a near miss or adverse event occurs? Consider whether you feel supported in reporting such an event, whether you believe your report will be followed up and how lessons are shared.*
4	*To what extent does mutual respect exist among professional groups (clerical staff, midwifery, neonatal nursing, obstetrics, paediatrics, anaesthetics, including trainees/students)?*
5	*What is your experience of the cohesion or otherwise among staff during an obstetric or paediatric emergency?*

All interviews were conducted by the same researcher (SVS), mostly during scheduled work hours. Two specialist medical staff were interviewed outside of work hours to avoid interruptions. Participants were asked to read the questions immediately before the interview and seek clarification where necessary. During each interview, the process of participant checks was employed. At appropriate times, the researcher’s understanding of the points made by the participants were rephrased by the researcher and the researcher’s understanding verified by the participant by his or her confirmation of the interpretation either verbally *(“that’s right”)* or non-verbally (“nodding”). Research bias was managed through the use of prolonged engagement [29]. This method involved the establishment of the interview and allowing the participants sufficient time to discuss all the pertinent issues from their own perspective with the researcher. There were no time constraints involved with the interview. Themes that arose from the interviews were able to be explored at the time of interview and were also able to be explored with other participants in subsequent interviews. Interviews lasted between 20 and 90 minutes, most lasting 60 minutes. Discussions were audio recorded and transcribed by an independent source. Transcripts were checked for accuracy by the interviewer and de-identified then forwarded to other members of the research team.

### Peer debriefing

The method of peer debriefing involved frequent discussions of the research at length with colleagues not involved in the research with methodological expertise. They offered support and guidance about the methodology and subject matter. In addition the researcher enlisted another person to conduct an audit of the transcriptions. Through external auditing of the tapes the accuracy of documenting the transcription was verified and the correct meaning of the data was derived.

### Data analysis

Qualitative data were explored using NVivo9® software (QSR International Pty Ltd, Melbourne, Victoria, Australia). Thematic analysis was used to code the data and identify emergent themes. Data were initially coded into 54 categories, with 1–124 entries in each category (Table 3). These were recoded and distilled [32,33] yielding three emergent themes. Data analysis and interpretation was checked independently by the other two authors. Multiple strategies were used to maximize trustworthiness of interpretations including triangulation, member checking and peer debriefing, providing rich, thick descriptions of the data [32]. The independence of one of the three-member research team (WC), with qualitative research expertise, minimized researcher bias, although this is an inherent attribute of naturalistic interpretive inquiry.

**Table 3:** **Codes and emergent themes from the data**

**Initial codes**	**Clusters**	**Themes**
Acceptance, Accountability, Audit, Balance, Barriers, Blame, Boundaries, Capabilities, Caution, Clinical judgment, Clinical Review, Collaboration, Communication, Confidence, Consistency, Culture, Evidence, Expectations, Experience, Fear, Goals to aspire to, Governance, History, Intimidation, Judgmental, Lack of support, Leadership, Maturity, Midwifery versus medical (non-intervention versus intervention), Organizational structures, Ownership, Perceptions, Personalities, Power, Priorities, Recognizing risk, Relationships between staff, Reporting adverse events, Resistance, Respect, Rules, Safety, Silos, Size, Staffing, Supervision, Support, Systems, Tension, Training, Trust, Women’s choice, Work-force, Work-load.	Accountability, Audit, Balance, Capabilities, Clinical Review, Culture, Goals to aspire to, Governance, History, Leadership, Organizational structures, Safety, Size, Support, Systems, Training	Clinical governance
	Acceptance, Blame Caution, Judgmental, Maturity, Respect, Trust	Acceptance, Trust, Respect
	Collaboration, Communication, Fear, Perceptions, Personalities, Relationships between staff, Tension	Relationships
	Barriers, Boundaries, Confidence, Experience, Intimidation, Lack of support, Midwifery versus medical (non-intervention versus intervention), Ownership, Power, Resistance, Silos, Women’s choice	Midwifery dominance, power, protection
	Clinical judgment, Consistency, Evidence, Expectations, Priorities, Recognizing risk, Reporting adverse events, Rules, Staffing, Supervision, Work-force, Work-load	Rules

### Triangulation

Data triangulation comprises the use of many data sources with matching foci to obtain different views about a topic [34]. Data may be gathered at different times, at different sites or from different people [34]. In this study data triangulation was undertaken between interviewees and themes were cross-referenced from one to another. In this way themes that were unique to a single participant as well as themes that were common to all participants were identified. These emergent themes were synthesized and the process was repeated until there was minimal overlap in the remaining categories.

### Ethics considerations

Approval to undertake this study was granted by Southern Health Human Research Ethics Committee (HREC) and Monash University HREC. Informed voluntary consent was obtained from each participant.

## Results

Three principle themes arose from analysis: clinical governance, dominance of midwives, and inter-professional relationships. Two subthemes were also evident: rules (within the clinical governance theme) and acceptance, trust and respect (within the inter-professional relationships theme).

### Clinical governance

The existence and importance of a robust and visible clinical governance framework was identified as a dominant theme. Specifically, participants were able to describe the institution’s numerous mechanisms, processes and systems at the local, site and organizational level to improve patient safety and minimize patient harm. These included the existence and widespread use of evidence-based clinical practice guidelines, the roles and activity of a local Practice Improvement Committee (a clinical governance oversight committee), and the routine use of regular and formal practice review. Senior staff demonstrated a high level of understanding of these various components of the clinical governance framework, while junior staff demonstrated that they had an awareness of the existence of the various activities even if they could not describe the components in detail. All staff were confident that breaches of safety would be acted upon swiftly and appropriately with the focus being on improving systems rather than taking a punitive approach.

Example and illustrative staff quotes are detailed below.

*specialist obstetrician:* “… our safety is always paramount … because we are low risk … we always make sure that … we’re very careful to practice within … those guidelines … so that safety of the women comes first, … there is a work-force awareness of importance of safety and quality … there is engagement in a safety and quality framework”.

*nurse administrator:* “an incredibly proactive team, very open to learning, and trusting that mistakes will be learned from, we’ll learn from them, so always, always reported, always explored and investigated well locally, reported through the organizational channels through the quality coordinator and the Directors of Nursing and also via the site, through the PIC *(practice improvement committee)* for interdisciplinary conversation, where they really explore and try to tease out what the issues are, to understand because it’s usually always about systems. It’s very rarely about individuals and really understanding you know where the incident has come from and where the changes need to be made”.

### Rules

Participant adherence to organizational policies and procedures was strongly apparent. Midwives were perceived to have good knowledge of organizational procedures and to encourage adherence, which participants agreed enhanced the safety of the service. This is exemplified by the following quote from a specialist obstetrician “… midwives here … they really follow the protocol”.

In contrast, while obstetricians acknowledged the existence of organizational policies and procedures, and were aware of their content, there was a perception among senior medical staff that professional opinion was sometimes preferable:

*specialist obstetrician:* “… guidelines and protocols may not strictly apply to each and every situation because clinical scenarios can be quite different at times so you have to use your own judgment”.

### Dominance of midwives

Participants reflected on the strong influence that midwives had in how maternity care was provided. They commented on the dominant role of the midwife, particularly relative to junior medical staff, and the effect on the balance of power among members of the clinical team on any given shift. While midwifery dominance was perceived to be generally beneficial in ensuring adherence to organizational policies and procedures, concern was also expressed that the dominant role of the midwife may, at times, adversely affect the way some midwives interacted with the junior medical workforce. In particular, there was concern that, at times, midwifery dominance led to the exclusion of junior staff, both medical and midwifery, that limited the clinical experience and learning opportunities of the junior staff. Midwifery dominance was also reported to influence clinical care delivery and intervention or non-intervention. For example, several respondents made reference to a midwifery preference against the use of epidural regional anaesthesia for pain management in labour that, due to perceived dominance of senior midwives over junior medical staff, prevailed, often irrespective of patient preference. This perception is supported by the observation that, of the three Monash Health maternity services, this service has the lowest epidural uptake rate [35].

*specialist obstetrician:* “the reason the midwifery and the medical workforce get on well is that actually they both want the same thing; the midwifery workforce wants to get on, do their work in an independent manner, in a stand alone manner, and only call for obstetric assistance if it’s required. And they make that judgment; and the medical staff … actually don’t want to be involved in the service. They want to be involved as little as possible because they’ve got busy private practices and are happy to come in to do the odd you know lift out forceps or caesarean section”.

*senior midwife:* “students and residents (junior medical officers*)* feel that the midwives are far too bossy in their approach towards them”.

Participants described dichotomies between non-interventionist midwifery paradigms and medical interventions deemed unnecessary that might impact on processes of labour and birth. A number of cases were provided where midwives denied women requesting epidural analgesia in early established labour, as examples that some midwives worked according to their personal views of promoting normal non-interventionist labour and birth rather than what an individual woman might choose. Forty eight percent of interviewees commented on the behaviour of a few midwives, during the initial months of operation, of managing the care of labouring women in isolation and according to their personal midwifery practice principles rather than as per organizational procedures. In extreme cases, medical staff had been refused access to women by midwives perceived to be protecting women from unnecessary intervention, such as routine vaginal examination. References were made to nulliparous women having prolonged second stage of labour without appropriate assessment of fetal well-being or the need to expedite birth, the assumption being that birth is a natural process that should occur without intervention. The extent to which any individual woman might have been complicit with such behaviour from a midwife could not be explored by the study design and so was not investigated as part of this study. That almost half the participants made reference to these past practices suggests lingering negative perceptions about a midwifery practice that had supported an individual midwife’s beliefs rather than support women’s choice inclusive of all members of the healthcare team. These actions were perceived as contributing to potential breaches of safety, specifically where there was inadequate fetal monitoring or assessment of need for assisted delivery in women with a prolonged second stage of labour. Participants who made reference to such behaviours also pointed out that organizational strategies, such as refinement and reinforcement of practice protocols, had been implemented to ensure practice conformity and had resulted in midwives either modifying their behaviour or leaving the service. This outcome was perceived as improving relationships between remaining senior members of the clinical team.

*midwife in charge of shift (associate midwifery manager):* “In the early days, the midwives were very protective of their women and we … weren’t allowed in those rooms by those midwives, whereas these days now I know exactly what’s going on in all those rooms” and “there were midwives who were very possessive of these birthing women and were reluctant to let people in”.

*specialist obstetrician:* “in the early days, that’s where we had the midwives who used to want to overstep their boundaries and didn’t want to talk to the manager you know, but these days now we don’t have any problem with our midwifery staff, not at all”.

Nonetheless, overall midwives were seen, both by themselves and by senior and junior medical staff, as the principle professional group making care management plan decisions, as illustrated by the following quotes:

*junior medical officer:* “It is a midwife led unit so they, they are technically running this place, but it was unlike any other departments that I’ve worked in where we don’t have to ask the nurses, you know, any permission. Just the patient’s permission to go and speak to the patient, get a history, do an examination and everything. But here it felt like it was the other way around; where we must ask permission of, either the nurse in charge or the midwife to actually go and speak to the patient and I found that difficult, being the only doctor on the floor with a responsibility for the welfare of all the patients if something goes wrong ..… often they’re very protective of their women”.

### Inter-professional relationships

There was evidence that, at times, relationships between staff could be tense and could potentially impact on service delivery. A senior midwife reflected on the importance that relationships has on service delivery. “… you obviously have personality conflicts so you just have to get on with that, to step in where you need to …” and “…we’re very clear that though it is a midwifery led model we have certain guidelines that we have to follow, and we have to involve our consultants and our doctors and we get medical students coming through now you know, so we have to really involve them in the woman’s care as well, it’s not just midwives you know. So there’s not that same boundary push anymore as there, in fact we don’t have any of it anymore”.

Similarly, an obstetrician noted the importance of relationships and interdependence between obstetricians and midwives in maternity care: “you can’t do midwifery on your own, nor can you do private obstetrics on your own; you have to have midwife support”.

### Acceptance, trust, respect

Participants believed that, overall, there were good inter-professional relationships between the various health professions. A specialist obstetrician recounted the interdependence of midwifery and obstetrics and the need for mutual respect to run a safe and efficient service.

*specialist obstetrician:* “I have a lot of good things to say as far as mutual respect is concerned. Being a smaller place everyone’s quite friendly and they make you feel very comfortable to be with and then I think, we do respect each other for what we are, for each other’s opinions and yes there isn’t anything negative I can think. I would say everyone’s respectful”.

A quote from a senior midwife corroborates this perception: “they’re really good the obstetricians here, they will listen to what you say if you are disputing what they’re saying. But it’s not often, you know. They’re the ones calling the shots too, and so if you’ve given them the clinical information, they’re acting on what you’re saying because they respect you, they have respect for your knowledge about what’s meant to happen, about when you need them”.

However, among junior staff there was a sense that trust and respect needed to be earned. Two junior staff reported having to earn trust leading to an improved working relationship with midwifery staff.

“… at the very beginning to feel accepted was impossible, it was quite a challenge, emotionally draining and … they needed to gain trust in me as well”. This was corroborated by a specialist obstetrician in this quote: “midwifery staff … you know …. you really have to earn your stripes with them”.

## Discussion

In this study we set out to assess staff perceptions of safety and quality in a new low risk maternity service and to explore staff knowledge and involvement in clinical governance, including professional relationships within and between the different craft groups. While, reassuringly, staff reported that their maternity service was one of safety and quality, their perceptions identified opportunities for service improvement that may have broader applicability to other maternity services. Specifically, three key themes emerged – issues related to staff acceptance and involvement in clinical governance, including the uptake of and adherence to uniform policies and procedures, the dominance and influence of midwives within the service, and inter-professional relationships, particularly between senior and junior staff. These themes are consistent with the existing literature on the effect of team behaviours on patient safety [1-4,36,37] and have clear implications for the development and maintenance of a high quality and safe maternity service. Of course, our observations of the relationships between professional groups and the dominance of the midwifery workforce are really only of relevance to healthcare systems that have midwifery as an independent profession such as in Australia, New Zealand, and the United Kingdom.

Variation in clinical practice that is not justified is known to threaten patient safety [37] such that high performing healthcare systems seek to standardize clinical practice using evidence, where available, to guide that practice. This is the rationale behind the development and implementation of evidence-based clinical guidelines, or policies and procedures. However, the effectiveness of policies and procedures is dependent on clinician uptake [37], which in turn is influenced by clinician ego [37], and on a robust framework to monitor uptake and effect change. Indeed, one of the most common disruptive behaviours in the clinical workforce relates to non-compliance with organizational protocols [2,14,18,37]. In this regard, it is well recognized that policies and procedures cannot account for all clinical scenarios and that expert clinical judgment remains an important “overlay” to ensure that, where appropriate, care is individualized, even if this is apparently outside of the relevant policy [37]. In this study we found that, in the main, midwifery staff adhered to organizational policies and procedures more closely than medical staff, particularly senior medical staff. Indeed, only senior medical staff voiced a strong desire for individual clinician freedom. Unfortunately, we were unable to assess whether lack of adherence to protocols by senior medical staff was more often appropriate or inappropriate but we suggest that, at least in this setting, protocol violation is more likely to be a feature of obstetricians rather than midwives. This observation has relevance to effective implementation strategies for future protocols. Certainly, an audit of protocol adherence with maternity services would be a worthwhile routine quality measure, recording adherence by professional group and seniority.

That said, we also uncovered a concern among staff, both medical and midwifery, that when the service first opened some midwives did not follow organizational protocols. There was a perception that if the attending midwife was to be the woman’s guardian and advocate then she would need to breach hospital policy and that this was acceptable [24-27]. Participants reported that, at that time, the hospital identified this behaviour and implemented strategies to improve protocol compliance, and thereby build a perception of safety. Overall, this was seen as a positive response by the hospital. There is a clear challenge here for the hospital and its staff. It is recognized that there is a risk that rigid hospital policies can disempower women to the point of oppression [27], resulting in a service that is not “woman-centered”. Yet the desire for uniform agreed evidence-based practice is understandable. In this study, the staff reported that the enforcement of policy adherence led to the departure of midwifery staff who felt that they could no longer practice the type of midwifery that they thought they should be practicing [25-27]. For example, hospital policy required regular maternal and fetal assessment in labour, in accord with accepted clinical practice guidelines [3,38,39]. Some midwives believed that it was their role to protect women from such “unnecessary” intervention with some even viewing 4–6 hourly vaginal assessments in labour as assault. Study participants reported that these non-adherent behaviours prompted hospital interventions, including strict monitoring of adherence to protocol that in turn led some midwives to leave the hospital’s employment. Whether this enhanced or diminished service quality could not be assessed by our study but would be an important consideration for future work.

Nearly three quarters of adverse events in maternity care are related to failure in team processes and communication [2,19]. This emphasizes the importance of a highly functioning team as the foundation of a safe and effective service. The influence of teamwork on performance was demonstrated in a study of emergency response clinical teams [36] where the complexities in healthcare was shown to lead to team fragmentation, and thereby reduce team efficiency and productivity, and increase disharmony. To counter this, “artifacts” such as uniforms, equipment and tools to share information, have been suggested by some to be useful at creating cohesion between clinical team members [36]. Indeed, strong organizational hierarchy can be created with just uniforms, and how they were worn, with resulting impact on team dynamics, both positive and negative [36]. While traditional hierarchies were not apparent in our findings, separate midwifery and obstetric stereotypes were apparent and could be described as artifacts that influence cohesion or division among the team. Evidence of a workforce hierarchy was also apparent through the quite different responses given by senior and junior staff, regardless of their discipline. In particular, junior staff reported that they felt the need to earn trust and respect before being accepted and did not feel supported when they first join the clinical team. This was a subtle but important finding that made visible the perception that the senior workforce was less than accepting of inexperienced staff, only valuing junior staff once they were able to contribute to the team’s workload. This is important because it suggests that the senior workforce did not display an accurate understanding of the role of the trainee – that is to be trained – and the need to embrace and support junior staff in this training role rather than devaluing their contribution. We believe that this apparent lack of understanding is an important deficiency in our senior workforce that requires specific and targeted response. Implicit in the roles of both senior midwifery and medical staff is the requirement to train junior staff, both midwifery and medical. We believe that the findings of this study suggest that this expectation should be more explicit and that there are enhanced opportunities for senior staff of both craft groups to be involved in the formal and informal training of junior staff of both craft groups. In that regard, we recently introduced a simulation-based training program for undergraduate medical and midwifery students taught by senior obstetricians and midwives that has been highly regarded by all involved [40].

Applying group theory [9] to our findings suggests that this perceived disharmony and perhaps hostility within the team is likely to lead to disruptive behaviour, which in turn, would be expected to make adverse events more likely [21]. Targeted education of senior staff might be expected to improve the senior-junior dynamic and thereby improve team function, reduce disharmony and improve patient safety. This, of course, would need to be tested but is a promising opportunity to enhance patient safety. Certainly, future assessment of safety of a service would usefully explore the functionality of junior-senior interactions. Unfortunately, we were unable to explore whether the senior-junior tension was equally apparent in the midwifery and medical workforces and so it would be worth exploring the two workforces separately, particularly if one workforce could offer insights to the other.

An extension to, but quite separate from, the senior-junior tension was the theme of midwifery dominance. We had abundant references from junior medical staff reporting frustration at being ordered around by senior midwives, leaving them with feelings that their contribution to the workplace was irrelevant. This is not a finding unique to our service. Relationships between midwives and medical staff have been explored by others [21], with medical staff commonly reporting that midwives were disrespectful and argumentative, particularly towards junior staff, and that midwives often obstructed medical staff from gaining experience [21]. Nonetheless, until this study, we thought that relationships between medical and midwifery staff in our service were good. The tension between junior medical staff and midwives only became apparent through our open-ended, explorative, themed interviews. Of course, inter-professional tensions are not even limited to medical versus midwifery. In the US, tension between obstetric nurses and midwives has been explored [22]. Tensions between these two groups is very similar to that between midwives and obstetricians, where nurses’ and obstetricians’ practice principles are seen by midwives to medicalise what they perceive to be a normal physiological process that should not be interfered with unless medically indicated. Certainly our findings highlight the importance of exploring inter-professional relationships as a component of building and sustaining a safe and effective maternity service. Ensuring multidisciplinary involvement at all levels of the governance framework, including protocol development and implementation, case review, and outcome audits, would be expected to engage the craft groups equally and enhance relationships. This would be expected to all and to ensuring good collaborative relationships between the professions so that patient complications are dealt with if and when they arise, and yet intervention is not applied in the absence of complications [25]. “Authentic mutual regard and trust creates environments where care is not only of high quality, but continually improving, and where staff enjoy coming to work and working together” (Downe [25] p 291). Indeed, in workplaces with high levels of mistrust the opposite occurs. Negativity breeds mistrust and fuels disharmony among team members whose interests become focused on individual survival rather than optimising patient care [10,20-22,36]. Thus, our observations suggest that solutions to disharmony between midwifery and medical staff – a common observation – are likely to need consideration of levels of medical intervention, ensuring that this is appropriate. We believe that this would help grow a confident and trusting medical and midwifery workforce.

This might be difficult. Intervention without clinical indication is common [28]. In particular, obstetricians aged 40 years or younger appear to favour medicalised care, including elective caesarean section, compared to their older counterparts [28]. Most were in favour of increased midwifery care during labour and reduced induction of labour rates for women free of complications but most were opposed to homebirth. Some obstetricians reported that midwives withheld important clinical information to delay the obstetricians’ attendance; midwives reported using delay tactics to minimize unnecessary medical intervention. Such behaviour was evident in our study, including exclusion of senior midwifery input in the assessment of women in labour during the early years of the service. Participants stressed that these issues have since been resolved.

## Conclusions

Exploring staff perceptions of the safety and quality of their service has afforded important and unexpected insights into mechanisms of a less safe service and opportunities for a safer service. It is certainly obvious that there is a need to develop a better understanding of how teamwork impacts patient safety. Our study shows that while there was resounding affirmation of a safe and quality service there was clear evidence of impaired teamwork and team tensions – factors known to compromise patient safety. In particular, we found that senior staff appeared to have an unrealistic view of the role of junior staff and that senior midwifery staff were particularly hostile to junior medical staff. In a maternity setting midwives have arguably the greatest potential to influence staff morale and thereby team performance. We suggest that each of the emergent themes reported here offer opportunities for service enhancement. Specifically, practice improvement initiatives, such as increasing the involvement of all professional groups in service planning, review, and governance, might be expected to improve relationships and better balance authority. Future research could address such initiatives as a template for other services.
